# A Case of Negative Pressure Wound Therapy Following Open Window Thoracostomy With Muscle Flap Filling for Chronic Empyema With Bronchopleural Fistula

**DOI:** 10.7759/cureus.86964

**Published:** 2025-06-29

**Authors:** Daiki Hayashi, Kensuke Kojima, Kyoichi Okishio, Kazunari Tsuyuguchi, Hyungeun Yoon

**Affiliations:** 1 Department of General Thoracic Surgery, NHO Kinki Chuo Chest Medical Center, Osaka, JPN; 2 Department of Thoracic Oncology, NHO Kinki Chuo Chest Medical Center, Osaka, JPN; 3 Clinical Research Center, NHO Kinki Chuo Chest Medical Center, Osaka, JPN; 4 Department of Infectious Disease, NHO Kinki Chuo Chest Medical Center, Osaka, JPN

**Keywords:** bronchopleural fistula, chronic empyema, muscle flap transposition, negative pressure wound therapy, open window thoracostomy, single-stage surgery, tuberculous empyema

## Abstract

Negative pressure wound therapy (NPWT) after open window thoracostomy effectively reduces empyema cavity volume, but fistula control remains challenging in cases of bronchopleural/alveolopleural fistula. We report the successful treatment of chronic tuberculous empyema with bronchopleural and/or alveolopleural fistulas through primary closure during thoracostomy, combined with simultaneous NPWT. A woman in her 90s, with a history of left upper lobectomy (60 years prior), developed chronic tuberculous empyema with a cutaneous fistula. Chest computed tomography (CT) showed an air-containing empyema cavity with surrounding pneumonia. Despite spontaneous cutaneous fistula closure, thoracic drainage confirmed an air leak, and *Mycobacterium* *tuberculosis* was isolated. Open window thoracostomy was performed with fourth-ninth rib resection (20 cm incision), alveolopleural fistula coverage using a pedicled serratus anterior flap, and cavity volume reduction with latissimus dorsi and serratus anterior flaps. NPWT commenced on postoperative day 3, continuing for six weeks after negative foam cultures. The empyema cavity decreased by 88.8%, from 118.2 cm³ to 13.2 cm³, facilitating gauze-based wound care and enabling patient transfer on day 65. Primary fistula closure during open window thoracostomy enables early implementation of NPWT, facilitating marked cavity reduction in chronic tuberculous empyema with fistula and representing a novel therapeutic strategy.

## Introduction

Chronic empyema affects approximately 0.7% of hospitalized patients with pneumonia [1], and its treatment imposes significant resource utilization due to prolonged hospitalization, repeat surgeries, and complex wound care. Chronic empyema remains one of the most challenging conditions in thoracic surgery, and treatment strategy selection critically influences patient outcomes. In cases where complete resection of the empyema sac is not feasible, a two-stage surgery consisting of open window thoracostomy (OWT) for infection control, followed by muscle transposition or thoracoplasty, has been established as the standard treatment approach [2]. Thoracoplasty, defined as the surgical removal of multiple ribs to collapse the chest wall and obliterate the empyema cavity, has been traditionally used for intractable cases. Recently, negative pressure wound therapy (NPWT) has shown efficacy in post-OWT empyema cavity management through enhanced wound healing by generating mechanical tension that stimulates granulation tissue formation, reducing bacterial burden, and facilitating fluid removal [3]. Conversely, chronic empyema complicated by bronchopleural fistula (BPF) presents unique challenges, as persistent air leakage leads to prolonged infection and expansion of the empyema cavity, making fistula control crucial for successful treatment [4]. Traditional two-stage surgery requires extended periods from the initial OWT to definitive closure, resulting in diminished quality of life and increased healthcare resource utilization [5,6]. In this case, a single-stage muscle flap closure of the fistula with concurrent empyema cavity volume reduction was performed during OWT for chronic empyema with fistula. Postoperative NPWT was initiated simultaneously, resulting in a favorable clinical outcome. This single-stage therapeutic strategy potentially reduces hospitalization and represents an efficient healthcare delivery model. This report examined the treatment course and efficacy of this approach.

## Case presentation

A 91-year-old woman residing in a nursing facility (Eastern Cooperative Oncology Group (ECOG) Performance Status 1) with a history of left upper lobectomy for pulmonary tuberculosis at 30 years of age presented with fever and left-sided chest pain. She had developed chronic tuberculous empyema with a cutaneous fistula one year prior to presentation and received conservative management with antituberculous therapy. Her past medical history included hypertension and age-related osteoporosis, with no known history of diabetes mellitus, coronary artery disease, malignancy, or immunosuppressive therapy. She maintained a stable body weight and an adequate oral intake prior to admission. Cognitive function was intact, and she was able to independently perform basic activities of daily living such as eating and grooming. Chest computed tomography (CT) at the referring hospital revealed secondary pneumonia due to empyema with a fistula, prompting transfer to our institution for surgical intervention.

The patient measured 148 cm in height and weighed 43.3 kg, with a calculated body mass index of 19.7 kg/m². The patient appeared alert and fully oriented. Her vital signs were as follows: body temperature, 36.3°C; blood pressure, 138/64 mmHg; regular pulse, 86 beats/min; respiratory rate, 20 breaths/min; and oxygen saturation, 97% on room air. An old surgical scar was noted on the left chest with markedly diminished ipsilateral breath sounds. On chest examination, breath sounds were markedly diminished over the left hemithorax with dullness to percussion and mild tenderness along the lower intercostal spaces. No chest wall deformity or fluctuance was noted. The cutaneous fistula spontaneously closed. Antituberculous therapy with isoniazid (250 mg) and rifampicin (450 mg) is ongoing. 

A complete blood count showed the following: white blood cell count, 6,140/μL with 72% neutrophils (neutrophil predominance); hemoglobin, 11.2 g/dL (mild anemia); and platelet count, 339,000/μL (normal). Serum biochemistry revealed the following: total protein, 6.7 g/dL; albumin, 2.7 g/dL (hypoalbuminemia); and C-reactive protein, 2.8 mg/dL (mildly elevated) (Table 1). Acid-fast bacilli smear and nucleic acid amplification tests for *Mycobacterium tuberculosis* were positive in both sputum and pleural fluid samples, while no other clinically significant bacteria were identified.

**Table 1:** Baseline clinical parameters BMI: body mass index; CRP: C-reactive protein; ECOG: Eastern Cooperative Oncology Group; WBC: white blood cell

Parameter	Value	Reference range/interpretation
Age	91 years	−
Height/weight/BMI	148 cm/43.3 kg/19.7 kg/m²	Normal BMI: 18.5–24.9
ECOG performance status	1	Ambulatory, capable of self-care
Temperature	36.3°C	Normal
Blood pressure	138/64 mmHg	Normal
Pulse rate	86 bpm	Normal
Respiratory rate	20 breaths/min	Normal
SpO₂ (room air)	97%	Normal
WBC/neutrophils	6,140/μL/72%	Mild leukocytosis
Hemoglobin	11.2 g/dL	Mild anemia
Platelet count	339,000/μL	Normal
Albumin	2.7 g/dL	Hypoalbuminemia
CRP	2.8 mg/dL	Mildly elevated

**Figure 1:**
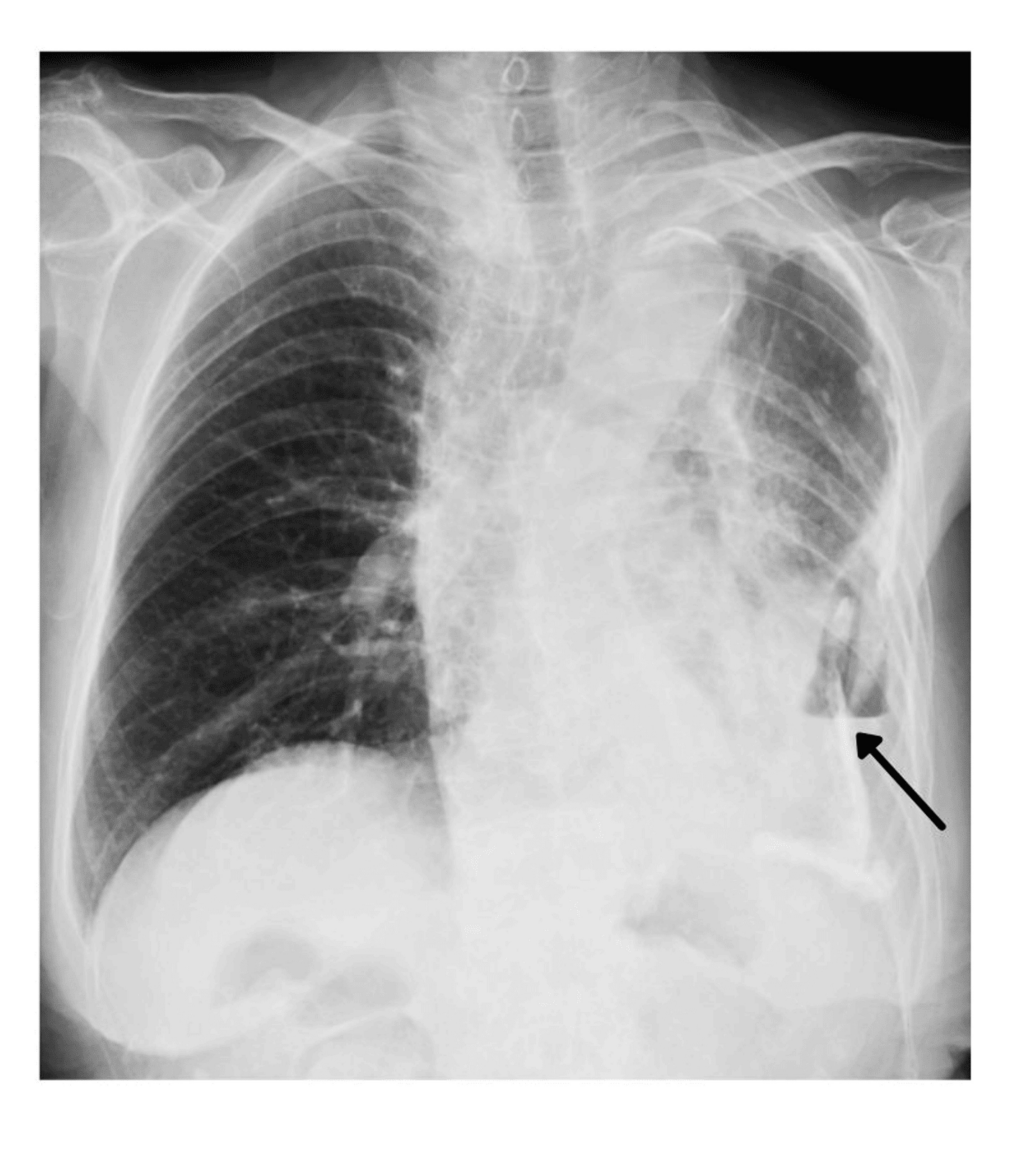
Chest radiography Chest radiography revealed fluid collection with an air-fluid level and calcification in the left mid-to-lower lung field, with adjacent areas of increased opacity suggestive of consolidation (black arrow)

**Figure 2:**
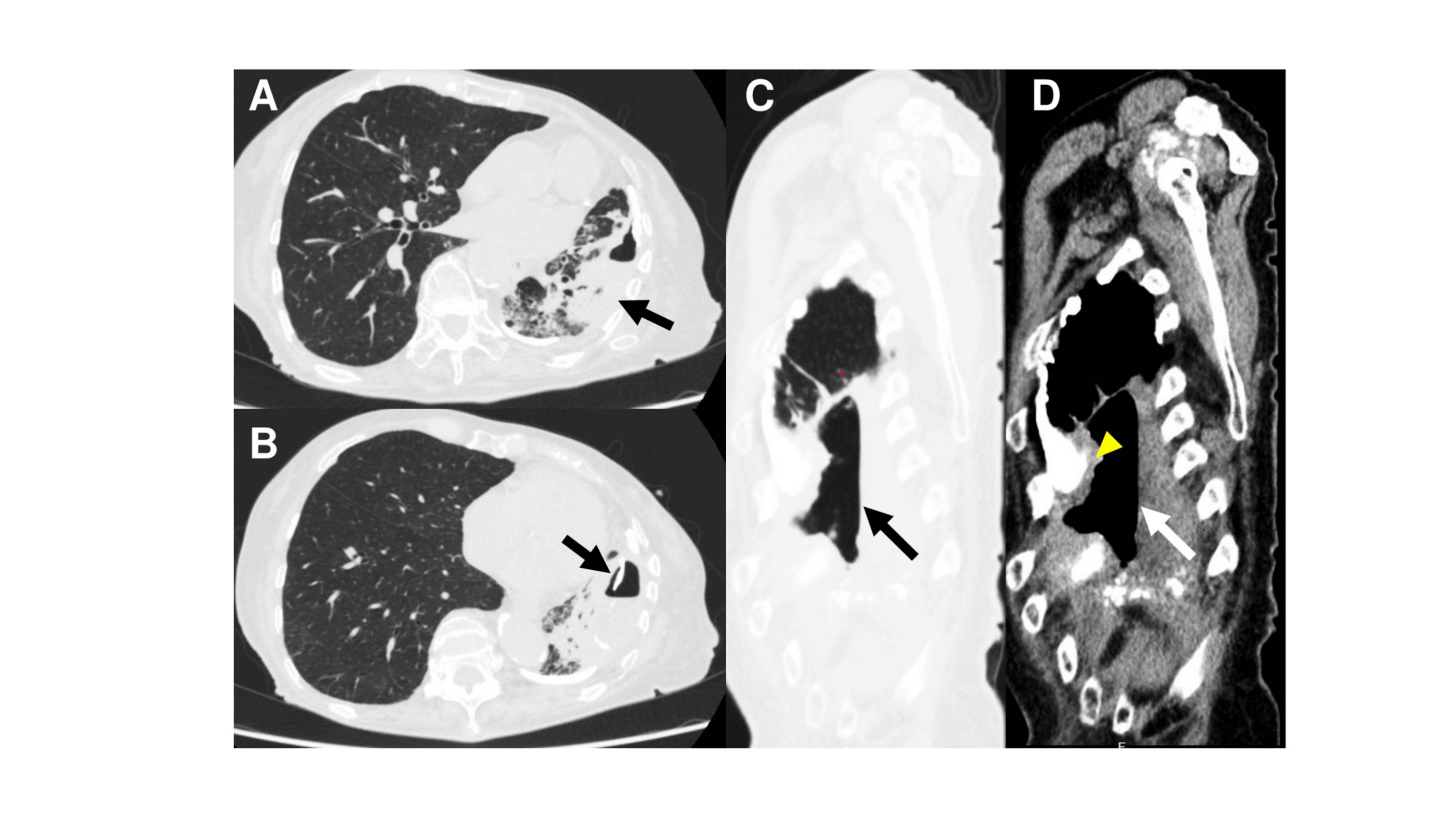
Preoperative computed tomography findings of chronic empyema with bronchopleural fistula (A) Axial computed tomography (lung window) demonstrating a left pleural empyema cavity containing air with surrounding pneumonic consolidation (black arrow). (B) Lung window showing extensive calcification within the site of fluid collection (black arrow). (C, D) Sagittal reformatted images in lung (C, black arrow) and mediastinal (D, white arrow) windows, respectively, revealing a craniocaudally elongated empyema cavity with focal intercostal space narrowing. In panel D, the markedly thickened pleural peel, indicative of chronic inflammatory remodeling, is highlighted by yellow arrowheads

Chest radiography demonstrated fluid collection with an air-fluid level in the left mid-to-lower lung field, accompanied by decreased perihilar lucency (Figure 1). Contrast-enhanced chest CT revealed a left pleural empyema cavity measuring 10 × 7 × 3 cm with a thickened pyogenic membrane of up to 10 mm, showing extensive calcification (Figure 2). The empyema cavity volume, as calculated by three-dimensional CT volumetry (SYNAPSE VINCENT®; Fujifilm Corporation, Tokyo, Japan), was 118.2 cm³. A BPF communicating with the residual left lower lobe was identified.

A 20 Fr thoracic drain was inserted through the ninth intercostal space at the posterior axillary line, yielding 300 mL of viscous yellow purulent fluid. Continuous air leakage was also observed. The patient reported progressive dyspnea, fatigue, and intermittent purulent sputum production over the preceding three weeks, with no significant clinical improvement under conservative management. Following thoracic drain placement, daily drainage volumes remained above 200 mL, and persistent bubbling indicative of a BPF was observed throughout the respiratory cycle. Serial chest radiographs showed no appreciable reduction in cavity size over seven days. These findings, coupled with the ongoing air leak, persistent infection, and lack of radiological improvement, prompted a multidisciplinary decision to proceed with surgical intervention. A single-stage OWT with muscle flap transposition and early NPWT initiation was planned to minimize hospital stay and enhance treatment efficacy.

**Figure 3:**
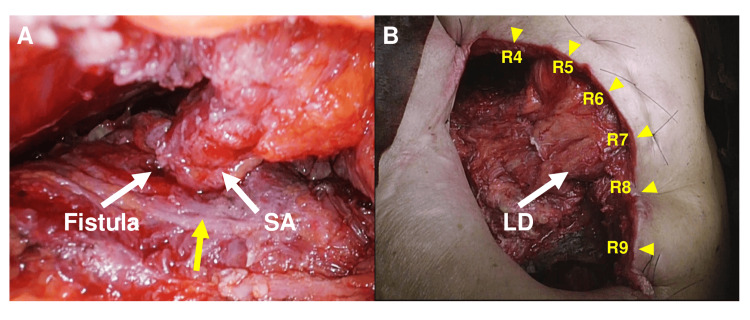
Intraoperative findings and surgical technique for single-stage open window thoracostomy with muscle flap transposition SA: serratus anterior muscle; LD: latissimus dorsi muscle (A) A bronchopleural fistula was identified at the cranial aspect of the fourth intercostal space (white arrow). The fistula was successfully sealed with a pedicled serratus anterior muscle flap (white arrow). The markedly thickened pleural peel, consistent with chronic inflammation and calcification, is also visible and highlighted by the yellow arrow. (B) A 20-cm posterolateral incision was made centered at the sixth intercostal space along the midaxillary line. Partial resection of the fourth through ninth ribs was performed to create the thoracostomy window. These rib levels are clearly indicated by yellow arrowheads with numerical labels (R4–R9) in the figure. After debridement of the calcified pyogenic membrane, the empyema cavity volume was reduced using latissimus dorsi and serratus anterior muscle flaps

Under general anesthesia in the right lateral decubitus position, a 20 cm posterolateral incision was made centered at the sixth intercostal space along the midaxillary line. Partial resection of the fourth through ninth ribs created a thoracostomy window. The pyogenic membrane, which was approximately 10 mm thick and highly calcified, was debrided using an ultrasonic scalpel. Two 5 mm BPFs were identified in the cranial aspects of the fourth and sixth intercostal spaces. A pedicled serratus anterior muscle flap was harvested and sutured to the pulmonary parenchyma surrounding the fistulas using absorbable monofilament sutures to achieve complete coverage (Figure 3A). The dead space was reduced using the latissimus dorsi and remaining serratus anterior muscle flaps (Figure 3B). The operative time was 5 hours and 32 minutes, with an estimated blood loss of 1030 mL.

**Figure 4:**
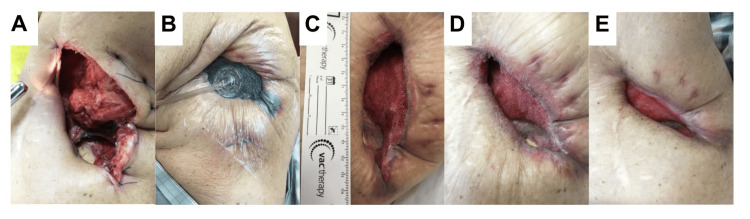
Postoperative course demonstrating progressive empyema cavity reduction with negative pressure wound therapy NPWT: negative pressure wound therapy (A) Immediate postoperative appearance following open window thoracostomy with muscle flap obliteration. (B) Postoperative day 3. Initiation of NPWT. (C) Postoperative day 32. The longitudinal diameter of the wound measured 11 cm, as confirmed by an adjacent sterile ruler included in the photograph for scale. (D) Postoperative day 43. Upon completion of NPWT, the superficial latissimus dorsi muscle flap demonstrated excellent granulation tissue formation. (E) Postoperative day 62. The empyema cavity volume had decreased and could only accommodate 20 mL of saline before overflow

After confirming the absence of rebleeding on postoperative day 1 (Figure 4A), NPWT (V.A.C.® Therapy System, KCI, San Antonio, TX, USA) was initiated on day 3 with a pressure of -50 mmHg (Figure 4B). The polyurethane foam was changed every 48-72 hours. The negative pressure was incrementally increased by 25 mmHg at each dressing change to a maximum of -200 mmHg. NPWT was continued for six weeks, the maximum duration covered by insurance reimbursement. To monitor microbial burden, bacteriological cultures of the foam were obtained at weeks 2 and 6 during dressing changes. All cultures remained negative for acid-fast bacilli throughout the course of treatment.

**Figure 5:**
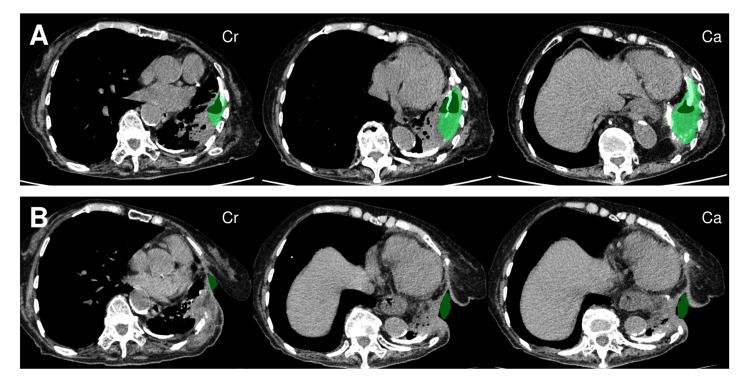
Three-dimensional volumetry demonstrating marked empyema cavity reduction following treatment Cr: cranial; Ca: caudal Comparison of the preoperative (A) and postoperative (B) computed tomography findings of the empyema cavity. Volumetric measurements were conducted using a three-dimensional reconstruction software program (SYNAPSE VINCENT®; Fujifilm Corporation, Tokyo, Japan) with semi-automated segmentation under the lung window setting (window width: 1500 HU, window level: -500 HU). The images are arranged in cranial-to-caudal sequence from left to right. Directional indicators "Cr" and "Ca" are shown in the figure to denote cranial and caudal orientation, respectively. Volumetric measurements revealed a dramatic 88.8% reduction in cavity volume from 118.2 cm³ to 13.2 cm³

Progressive empyema cavity reduction was observed after the initiation of NPWT (Figures 4C,4D). Three-dimensional CT volumetry at six weeks demonstrated marked cavity reduction to 13.2 cm³ (11.2% of the initial volume) (Figure 4E, Figure 5). The wound cavity was sufficiently small to accommodate only 20 mL of saline, enabling management with conventional gauze dressing changes. The patient was transferred to a local hospital on postoperative day 65. Pain was effectively controlled with scheduled acetaminophen during the initial postoperative week. At the 6- and 12-month outpatient follow-up visits, no major complications occurred, and no recurrence of empyema or fistula was observed, with successful management maintained through twice-weekly gauze changes at the thoracostomy site.

## Discussion

We achieved favorable outcomes in chronic empyema with BPF through a single-stage treatment combining OWT, immediate muscle flap closure, and early implementation of NPWT. Three-dimensional CT volumetry demonstrated an 88.8% reduction in the empyema cavity within six weeks, achieving a substantial reduction in treatment duration in comparison to conventional two-stage approaches. Thus, this strategy may be an effective therapeutic option for appropriately selected patients.

The standard treatment for chronic empyema follows a two-stage approach: initial OWT for drainage and infection control, followed by months of open wound management before definitive closure with muscle transposition or thoracoplasty [7]. However, this strategy presents inherent challenges, including prolonged treatment and an increased risk of secondary infection at the thoracostomy site. While previous reports on NPWT in empyema treatment describe a 60% cavity volume reduction, most involve the application of NPWT after OWT alone, with limited experience combining muscle flap obliteration [8].

The novelty of our approach lies in immediate fistula closure during single-stage surgery with early NPWT. Although conventional methods preclude the initiation of NPWT due to persistent fistula, we achieved the safe application of NPWT by postoperative day 3 through complete fistula closure during the initial procedure. This resulted in an 88.8% reduction in the cavity volume, exceeding previously reported outcomes, with healing achieved within approximately two months, enabling a dramatic reduction in the treatment duration. Furthermore, three-dimensional CT volumetry provides a quantitative assessment that complements traditional subjective evaluation methods.

Biologically, the synergistic effects of muscle flap obliteration and NPWT appear to be fundamental to treatment success. Pedicled muscle flaps maintain tissue viability through stable vascular pedicle perfusion, contributing to infection control and wound healing via sustained local delivery of antimicrobial substances and growth factors [3,9,10]. Concurrently, NPWT promotes the formation of granulation tissue, a reduction in bacterial burden, and mechanical contraction of the cavity through continuous negative pressure [11]. We hypothesize that immediate reduction of the dead space using muscle flaps combined with NPWT-enhanced wound contraction creates an optimal microenvironment for accelerated healing.

This study has several limitations. First, as a single-case report, the findings may not be generalizable; additional cases are required to validate the broader applicability of this approach. Second, clear criteria for patient selection must be established. Although our patient maintained a relatively good nutritional status despite her advanced age, this strategy may not be suitable for individuals with severe malnutrition, ventilator dependence, or extensive bilateral disease. Third, long-term follow-up is necessary to assess the durability of treatment effects and recurrence risk. Finally, the interobserver variability of volumetric measurements was not formally assessed in this case. Future studies should incorporate reproducibility testing to validate this quantitative imaging modality as a reliable outcome measure.

## Conclusions

This single-stage strategy for chronic empyema with BPF may offer a promising alternative to the conventional two-stage approach in selected patients. By combining immediate BPF closure with muscle flap transposition and NPWT, this method achieves rapid cavity closure, minimizes treatment duration, and avoids prolonged thoracostomy-related morbidity. Compared to traditional staged procedures, this approach may have the potential to enhance therapeutic efficiency, particularly in the most challenging cases complicated by BPF. Early social reintegration and optimized healthcare resource utilization are additional benefits. Multicenter prospective studies with long-term follow-up are warranted to establish this as a standard treatment modality.

## References

[1] Ahmed RA, Marrie TJ, Huang JQ (2006). Thoracic empyema in patients with community-acquired pneumonia. Am J Med.

[2] Molnar TF (2007). Current surgical treatment of thoracic empyema in adults. Eur J Cardiothorac Surg.

[3] Shen KR, Bribriesco A, Crabtree T (2017). The American Association for Thoracic Surgery consensus guidelines for the management of empyema. J Thorac Cardiovasc Surg.

[4] Lois M, Noppen M (2005). Bronchopleural fistulas: an overview of the problem with special focus on endoscopic management. Chest.

[5] Garcia-Yuste M, Ramos G, Duque JL (1998). Open-window thoracostomy and thoracomyoplasty to manage chronic pleural empyema. Ann Thorac Surg.

[6] Shamji FM, Ginsberg RJ, Cooper JD (1983). Open window thoracostomy in the management of postpneumonectomy empyema with or without bronchopleural fistula. J Thorac Cardiovasc Surg.

[7] Massera F, Robustellini M, Della Pona C, Rossi G, Rizzi A, Rocco G (2009). Open window thoracostomy for pleural empyema complicating partial lung resection. Ann Thorac Surg.

[8] Nishii K, Nakajima T, Yamamoto T (2021). Management of thoracic empyema with broncho-pulmonary fistula in combination with negative-pressure wound therapy. Gen Thorac Cardiovasc Surg.

[9] Arnold PG, Pairolero PC (1990). Intrathoracic muscle flaps. An account of their use in the management of 100 consecutive patients. Ann Surg.

[10] Werner S, Grose R (2003). Regulation of wound healing by growth factors and cytokines. Physiol Rev.

[11] Argenta LC, Morykwas MJ (1997). Vacuum-assisted closure: a new method for wound control and treatment: clinical experience. Ann Plast Surg.

